# Development of a biorelevant dissolution method for inhalation products: Proof of concept using drugs with diverse solubilities

**DOI:** 10.5599/admet.2861

**Published:** 2025-09-04

**Authors:** Amar Elezović, Sandra Cvijić, Saša Pilipović, Alisa Elezović, Jelena Parojčić

**Affiliations:** 1 Control Laboratory of Agency for Medicinal Products and Medical Devices of Bosnia and Herzegovina, Maršala Tita 9, Sarajevo, Bosnia and Herzegovina; 2 University of Belgrade – Faculty of Pharmacy, Department of Pharmaceutical Technology and Cosmetology, Vojvode Stepe 450, Beograd, Serbia; 3 University of Sarajevo – Faculty of Pharmacy, Department of Pharmaceutical Technology, Zmaja od Bosne 8, Sarajevo, Bosnia and Herzegovina

**Keywords:** Inhalers, pressurized metered dose inhalers, dissolution, *in vivo-in vitro-in silico* correlation

## Abstract

**Background and purpose:**

Due to their unique application and action, inhalation products require specific quality tests, such as Uniformity of Delivered Dose and Aerodynamic Assessment of Fine Particles. While there's no current official requirement for dissolution tests, new draft guidelines are introducing them as a supportive or required measure; however, a universally accepted methodology for such testing remains elusive. The aim of the present study was to explore the discriminatory ability and *in vivo* predictability of the newly developed dissolution assembly.

**Experimental approach:**

The applied experimental approach to biopharmaceutical characterization of inhalation products involved developing a biorelevant method for testing the dissolution rate of the selected active substances. Seven commercially available products, formulated as pressurized metered dose inhalers, containing either salmeterol xinafoate or beclomethasone dipropionate, have been studied. The research strategy combined in vitro testing within silico simulations.

**Key results:**

The developed dissolution method did not detect significant differences in the case of products containing highly soluble salmeterol, but it did reveal differences for products containing poorly soluble beclomethasone dipropionate. Moreover, a correlation was identified between the dissolution test results and absorption constants for beclomethasone dipropionate.

**Conclusion:**

The obtained results indicated that the investigated products would not be considered bioequivalent based on the aerodynamic particle size distribution. It was demonstrated that a discriminative dissolution method can be developed through a well-established paradigm of dissolution testing, while taking into account the specificities of the inhalation route of administration.

## Introduction

Inhalation products are, in many ways, specific pharmaceutical products. The method of application and the place of action determine the particularity, both in the technological and analytical sense. These specificities require specific quality tests that ensure the performance and quality of a drug product. Specific tests, along with the necessary equipment for their implementation, enable control over the quality parameters of inhalation preparations, which are, on the one hand, specific to these dosage forms and, on the other hand, important indicators of the medicine's quality and effectiveness. The list of mandatory quality parameters for testing inhalation products is given in the Guide for Pharmaceutical Quality of Inhalation and Nasal Preparations issued by the European Medicines Agency (EMA) [1]. Two tests specific to inhalation products that form the backbone of their quality control are Uniformity of delivered dose (UDD) and Aerodynamic assessment of fine particles (AAFP) [2]. Since preparations for inhalation are combination products, these tests examine the relationship and functionality of the medicinal preparation as well as the delivery device. UDD is a test that measures the amount of active substance released from the inhaler during activation or inhalation. AAFP examines the distribution of aerosol particles based on their aerodynamic diameter. Tests and procedures are described in the corresponding monographs of the European Pharmacopoeia [2].

According to the current EMA guideline [1], there is no requirement or mention of a dissolution test for preparations intended for inhalation. The dissolution test is introduced in the draft EMA guideline for the quality of inhalation products [3], where it is stated that “development and characterization studies based on dissolution testing can be provided as supportive information” for the pharmaceutical development and that “changes that affect the in vitro APSD or in vitro dissolution release characteristics of the finished product may be considered to have a significant impact”. A similar approach is suggested in the guidelines for specific drug products issued by the FDA. However, in the guidelines currently in effect, there are no requirements for conducting the test, which aligns with the conclusion of the 2008 Ad Hoc advisory panel for the USP performance test of Inhalation Dosage Forms [4]. In 2024, the revised draft guidance on budesonide and formoterol fumarate [5] and draft guidance on fluticasone propionate [6] were published. These guidelines require a dissolution test as one of the seven tests to demonstrate the in vitro equivalence of inhalation products. Although the draft guidelines provide recommendations for selecting the apparatus for conducting the test and evaluating the results, there is still no generally accepted methodology for conducting and evaluating the dissolution test results for inhalation products [7-9]. Numerous authors and research groups have developed models for testing the release rate of medicinal substances from inhalation preparations. The applied approaches can, in general, be described as those in which the dissolution of the entire preparation is tested and those in which the dissolution of particles previously classified based on their aerodynamic diameter is tested. Both approaches have advantages and disadvantages. Another way to differentiate the methods of testing drug dissolution rates from inhalation products is based on the technical performance of the apparatus used. The described procedures are based on the use of diffusion cells [10-13], Transwell systems [14-16], flow cells [17-19], modified USP apparatuses 2 and 5 [9,20], and proprietary systems based on diffusion cells [21-23]. Of the mentioned procedures, only a small number have been reported to be able to detect differences in formulations with the same active substance [12,16,20]. A more comprehensive overview of the published literature can be found in the relevant literature [7,24-26].

The aim of the present study was to explore the discriminatory ability and *in vivo* predictability of the newly developed dissolution assembly [27]. Seven inhalation products with marketing authorization containing either high or low solubility model drugs have been investigated.

## Experimental

### Materials

Beclomethasone dipropionate (BDP) and salmeterol xinafoate (SX) were chosen as model substances, and Pharmacopoeial reference standards (European Pharmacopoeia Reference Standards) were used for analysis. Seven pressurized metered dose inhalers (MDI) products containing BDP (3 preparations marked B1, B2 and B3) and SX (4 preparations marked S1, S2, S3 and S4) were tested. Inhalers were purchased on the markets of Bosnia and Herzegovina and Germany. All used chemicals were PA grade or better.

### Aerodynamic assessment of fine particles

Aerodynamic assessment of fine particles was determined in accordance with Ph. Eur. monograph 2.9.18 using the Next Generation Impactor (NGI) (Copley Scientific Limited, UK). Fine particle fraction (FPF), mass median aerodynamic diameter (MMAD), and geometric standard deviation (GSD) were calculated using the CITDAS v. 3.10 software (Copley Scientific, Nottingham, UK, https://pv-systems.pl/en/products/copley-scientific/19-inhaler-testing/ancillaries/100-copley-inhaler-testing-data-analysis-software-citdas).

### Solubility determination

The excess amount (around 100 mg) of the investigated active substances was placed in a 2 mL glass vials, Then 2 mL of the test medium was added, and the mixture was thermostated at 37 °C with constant stirring, using a Reacti-Therm^TM^ heating/stirring module (Thermo Fisher Scientific Inc., USA) for a minimum of 16 hours. After the predetermined time, the suspension was centrifuged at 14,000 rpm for 10 minutes, and then the supernatant was filtered through a nylon filter with a pore size of 0.22 μm. After appropriate dilution, the sample solutions were assayed for BDP/SX using the previously published HPLC methods [28,29].

### Dissolution test assembly

The developed method for testing the dissolution rate of inhalation preparations is based on the use of an abbreviated Andersen Cascade Impactor (aACI) (Andersen Cascade Impactor, Erweka GmbH, Germany) and a modified paddle-over-disc apparatus (Varian VK 7010, Varian Inc., USA).

**Figure 1.**
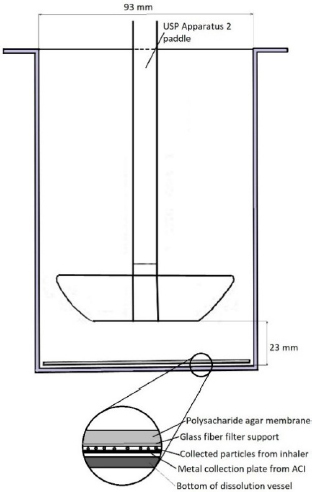
Presentation of the experimental setup for testing the dissolution rate of the active substance from the inhalation preparation [27]

The paddle-over-disc apparatus (USP App. 5) was chosen as the basic instrument for developing the method. The detailed dimensions of the assembly are given in Figure 1.

The membrane for the dissolution test assembly consisted of agarose gel prepared as a 2% agar gel in a dissolution medium (phosphate-buffered saline (PBS) with or without 1% sodium dodecyl sulphate (SDS). Agar and SDS were mixed with PBS and heated to 80 °C. A quantity of 4.5 mL of the mixture was poured into the recess on the back of the ACI plate and evenly distributed over the surface. A glass fiber filter was immersed in the still liquid solution and allowed to soak and sink into the agar. The plate was then transferred to moist tissue paper and covered with a Petri dish to allow the agar to solidify and gel without drying out.

### Dissolution test procedure

Samples for dissolution testing were obtained using the aACI. Using the arrangement of stages 0, 1, 2, 7 and the filter stage, the fraction of particles with an aerodynamic diameter ranging from 4.7 to 0.2 μm was collected.

After the inhaler doses were collected on the membrane, it was inverted onto the back of the second plate of the cascade impactor, carefully pressed to remove air from under the membrane, and fixed with aluminium clips. This system was immersed in a flat-bottom dissolution vessel with a 1 L volume and a diameter of 93 mm. The vessel was placed in a dissolution tester with a rotating paddle, positioned 23 mm from the bottom of the vessel. 300 mL of dissolution media, heated to 37.5 °C (PBS without SDS for SX or with 1% SDS for BDP testing), was used. The speed of the paddle rotation was set to 100 revolutions per minute (rpm). At the specified time intervals, sampling was performed with a probe along the edge of the vessel at a height of 25 mm from the bottom of the vessel and 5 mm from the wall of the vessel. The sample volume was 1 mL, and after each sampling, the same volume of fresh medium was added to the vessel.

Experimentally obtained dissolution profiles were evaluated using model-dependent methods (zero-order, first-order, Higuchi, and Hixon-Crowell models). Comparison of drug dissolution profiles from different products was made using model-independent methods (difference factor - *f*1 and similarity factor - *f*2). Data analysis was performed using Microsoft® Excel®.

### Design of in silico models

In silico compartmental models of the absorption, distribution, and elimination of the model drugs in the human body were created using the GastroPlus® software package (v. 9.8, Simulations Plus Inc., Lancaster, USA, https://www.simulations-plus.com/software/gastroplus/). Available data from the literature were used to create a two-compartment pharmacokinetic model in order to describe the intravenous and oral administration of BDP. Predicted plasma concentration-time (*c*_p_-*t*) curves of BDP and its main active metabolite, beclomethasone 17-monopropionate, were compared with published *in vivo* data [30] to confirm the validity of the models. The models were further expanded to account for drug absorption through the lungs, and for this purpose, the Pulmonary Compartmental Absorption and Transit (PCAT™) module of GastroPlus® was used. Parameters specific to each product included the percentage of drug deposition in the respiratory tract, which was calculated from data on the aerodynamic assessment of fine particles using a procedure published elsewhere [31]. Data from published pharmacokinetic studies of tested products [32,33] were used to optimize and validate the pulmonary in silico model.

## Results and discussion

The composition of the investigated products and their experimentally determined aerodynamic properties (MMAD and FPF) are presented in Table 1.

**Table 1.** Overview of the composition and characteristics of the investigated inhalation products

Inhalation product	Active substance	Declared dose, μg	Propellant	Modifier	Active substance 2	MMAD, μm (GSD)	FPF
S1*	Salmeterol xinafoate	25	norflurane	-	fluticasone propionate	3.042 (2.154)	0.53546
S2*		25	norflurane	-	-	1.865 (1.648)	0.68932
S3*		25	norflurane	-	fluticasone propionate	3.415 (1.669)	0.40170
S4*		25	norflurane	anhydrous ethanol, soy lecithin	-	1.535 (1.493)	0.49371
B1**	Beclomethasone dipropionate	100	norflurane	ethanol	-	0.880 (1.721)	0.69888
B2**		100	norflurane	ethanol	formoterol fumarate	1.028 (1.881)	0.57701
B3**		250	norflurane	anhydrous ethanol, oleic acid	salbutamol sulphate	1.683 (2.097)	0.56342

*S1, S2, S3 and S4 – commercial inhaler products containing SX

**B1, B2 and B3 – commercial inhaler products containing BDP

**Figure 2.**
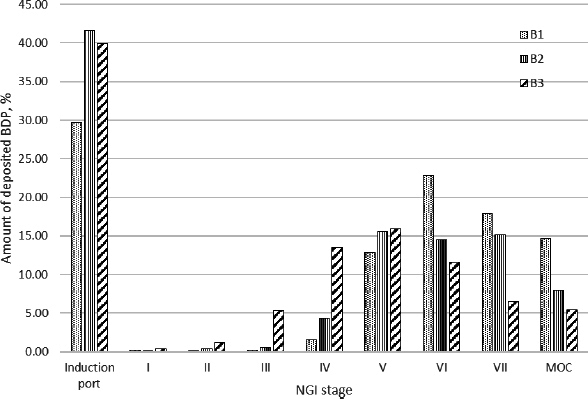
Distribution of aerodynamic size of BDP particles

**Figure 3.**
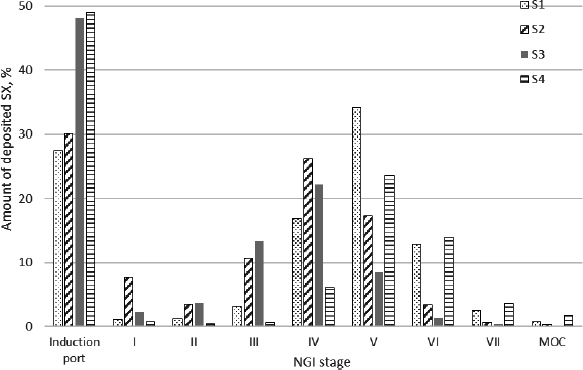
Distribution of aerodynamic size of SX particles

The results of the determination of the aerodynamic particle size distribution of the tested products containing BDP and SX, obtained using the NGI, are presented in Figures 2 and 3, respectively.

When observing the results obtained from testing products containing BDP, it is noticeable that the highest proportion of fine particles (FPF) was in preparation B1, while the other two products had similar values of fine particle fraction. The difference was also noticeable in the MMAD values (Table 1), which ranged from 0.880 μm in product B1 to 1.028 μm in product B2 and 1.683 μm in product B3. This, along with the differences in geometric standard deviation (GSD), aerodynamic particle size distribution (Figure 2), and the resulting dispersion of aerodynamic particle size, suggests potential variations in the deposition of drug particles in the respiratory tract among different inhalation products.

**Table 2.** Experimentally determined amounts of the model drugs deposited on the agar membrane in aACI stages 2-7

Sample	Amount of deposited active substance per dose, μg
B1	27.126
B2	21.809
B3	32.785
S1	7.099
S2	9.131
S3	5.563
S4	9.593

The differences in MMAD and GSD were more pronounced in SX than in BDP products (Table 1). Based on the MMAD values, it can be assumed that SX particles will be deposited in the upper parts of the respiratory tract, and the GSD values indicate that the differences in the “width” of the distribution between the four tested products will be more significant than the differences among the products containing BDP. In contrast to the products containing BDP, all four SX products had the same declared content value per dose (25 μg). Still, the differences in FPF were more pronounced. The results of the aerodynamic assessment of fine particles, according to the EMA guideline [34], indicate that the tested products would not be equivalent.

In the development of a biorelevant method for drug dissolution from a preparation for inhalation, the following critical factors affecting drug dissolution rate were carefully considered: (a) aerodynamic particle size distribution and the proportion of the respirable fraction of the drug; (b) type and volume of the test medium; (c) solubility of the drug substance in dissolution medium; (d) characteristics of the membrane through which the substance is absorbed; (e) device design; and (f) paddle stirring rate during the analysis.

In the published papers dealing with dissolution method development for inhalation products [9-11,14, 15,17-21,23], two approaches for sample collection are described: with or without separation of particles based on their aerodynamic diameter. Sampling without particle size separation is more straightforward and faster. However, such a sample does not represent a situation equivalent to what happens *in vivo*. Therefore, the dissolution testing of samples that are not separated into fine and coarse fractions may lead to results that do not accurately correspond to the *in vivo* situation. This is particularly true for dry powder inhaler (DPI) products with a higher proportion of large particles, which may be retained in the mouth and pharynx. Drug particles deposited in these regions are likely to be swallowed, dissolved, absorbed, and metabolized as in the case of oral preparation. Considering notable differences in drug absorption and elimination between oral and inhalation routes of administration, it may be argued that data on the dissolution of large particles does not provide an accurate estimate of the inhalation product's bioperformance. Accordingly, samples consisting of the fine particle fraction, which was considered to be deposited in the lower parts of the respiratory tract, were used for dissolution testing. This fraction is considered to consist of particles smaller than 5 μm. However, particles smaller than 1 μm are often not included in this fraction because they are considered too small to be retained in the lungs and are mostly expelled from the lungs through exhalation [35]. The ACI does not have stages that correspond precisely to these diameters. The closest stages are Stage 2, with a cut-off value of 4.7 μm, and Stage 7, with a cut-off value of 0.7 μm. Therefore, the ACI was assembled in an abbreviated version (aACI), with the stages in the following order: 0, 1, 2, 7, and the Filter stage. This setup allows particles in the aerodynamic diameter range of 4.7 to 0.7 μm to be retained on the surface of the plate below Stage 7. This range corresponds to the particle size values that are thought to be deposited and absorbed in the lower respiratory tract.

To calculate the fraction of the dissolved drug, it is necessary to know the amount of drug available for dissolution. This information is readily available in the case of oral products - the entire administered/ /declared dose is available for dissolution and subsequent absorption. The situation with preparations for inhalation is much more complicated. While one of the primary physiological functions of the gastrointestinal system is the transfer of nutrients, water, and electrolytes from the digestive tract into the bloodstream (*i.e.* absorption), the respiratory system's role is to prevent penetration and accelerate the removal of airborne particles. Specific pharmaceutical-technological solutions to these problems lead to a discrepancy between the declared dose and the amount of drug available for dissolution and absorption. The declared dose is a theoretical dose that has been previously measured or is being measured by the inhalation device. During activation, the entire dose does not leave the device; the portion that leaves the device and becomes available for inhalation is designated as the delivered dose. Additionally, the entire delivered dose does not reach the site of absorption. It is a common opinion that particles with an aerodynamic diameter of less than 5 μm reach and are deposited in the distal parts of the respiratory tract, where they can act locally or be absorbed. Therefore, the FPF of the inhalation products was selected as the target sample for the release rate test. Abbreviated ACI was used for its collection, and the fraction between stages 2 and 7 was collected. The obtained results are presented in Table 2. The obtained values were used to calculate the proportion (%) of the dissolved drug during the dissolution test.

The choice of a medium is a critical parameter for designing a dissolution test. The selected medium should simulate the physiological conditions and composition of the human body fluids, but on the other hand, it should be simple, stable and easy to use. After being deposited in the respiratory system, the active substance first comes into contact with the lung fluid, in which it must dissolve to be available for absorption and systemic or local action. Lung fluid plays a crucial role in maintaining the structure and function of the respiratory system. With its composition and surface tension effects, it directly stabilizes the structure and prevents the collapse of the respiratory system, mediates the transfer of gases between inhaled air and blood, and, through alveolar macrophages, leukocytes, and secreted antimicrobial substances, forms the basis of the body's defence system in the lungs [36-38]. Its composition varies depending on the location in the respiratory system. In the upper respiratory tract, the lung fluid layer forms a mucous gel 5 to 100 μm thick, whereas in the lower respiratory tract, the layer is less viscous, thinner (0.1-0.2 μm), and rich in surfactants [37]. Based on the previous considerations, it can be concluded that the “target” fluid for in vitro dissolution testing of an inhaled drug is not simulated lung fluid [39], but rather a fluid whose properties resemble those of the interstitial fluid and is easily prepared using standard, readily accessible chemicals. Isotonic phosphate buffer (phosphate buffer saline – PBS), often used in biochemical analyses and research, was chosen because it is isotonic, has a physiological pH (7.4), and osmolality and ion concentrations similar to those of human physiological values. PBS is stable, has a good buffer capacity, and is physiologically compatible. When selecting an appropriate dissolution medium, it is often recommended to ensure that sink conditions are met. Sink conditions are met when the medium volume can dissolve at least three times the drug dose used in the test [40]. The use of physiological surfactants present in the lung is not practical due to the complex procedure required for medium preparation and their limited availability. Therefore, surfactants commonly used for dissolution testing of oral solid dosage forms were selected: SDS and polysorbate 80. The experimentally obtained solubility of SX in PBS was 0.155 mg mL^-1^. This solubility allowed sink conditions to be achieved without the addition of surfactants to the medium; therefore, PBS was used as the test medium for SX. BDP is poorly soluble in PBS (solubility in water is 2.1 μg mL^-1^ [41]) and requires the addition of surfactants to achieve sink conditions. The solubility of BDP in solutions of SDS and polysorbate 80 in PBS was tested. The obtained results are presented in Table 3.

**Table 3.** Experimentally determined solubility of BDP in different solvents

Solvent	Solubility, μg mL^-1^
PBS	2.96
1 % polysorbate 80 in PBS	9.64
0.5 % SDS in PBS	48.96
1 % SDS in PBS	93.16

The obtained results indicate that BDP exhibits sink conditions in 0.5 and 1 % SDS solutions, as solubility exceeds three times the maximum theoretical concentration of BDP in the medium during the dissolution test (10 μg mL^-1^). 1 % SDS solution was chosen as the medium, providing the robustness for testing the release rate of inhalation products with BDP.

Membranes are an integral part of all systems described so far for testing the release rate of inhalation products [9,14-21,24,42,43]. Membranes made of polycarbonate, Teflon®, various cellulose derivatives or nylon have been used. The primary role of the membrane in these experiments is physical retention of particles and testing the influence of the membrane on the dissolution process itself was not the focus of these studies. Considering the choice of dissolution medium, which should simulate extracellular fluid and plasma, the largest and thickest membrane between the space filled by this fluid and the site of deposition of active substance particles is the mucous membrane. That membrane comprises 97 % water and 3 % dry matter (mucins, non-mucin proteins, salts, lipids, and cellular debris) [44]. The membrane for the dissolution test, in addition to being readily available and easy to prepare, should be mechanically stable and have a composition and porosity similar to those of the mucous membrane of the respiratory tract. A membrane made of agar, although not corresponding in composition to that of the mucous membrane in the lungs, meets all other requirements. Mechanical strength can be achieved by incorporating glass fiber filters into the membrane structure. In this way, a polysaccharide membrane with a standardized composition and structure was obtained, exhibiting satisfactory mechanical properties that enable necessary manipulation without compromising the physical integrity of the membrane. This membrane was stable and insoluble in the dissolution medium.

The principle of the paddle over disc apparatus (USP App. 5) was chosen as the basis for the development of the method. Sample processing and placement in other reported dissolution systems is more complicated and subject to greater errors due to the larger number of sample preparation steps, each of which introduces additional measurement uncertainty, and the use of small and/or non-standard volumes that introduce a larger relative error. Additionally, in other systems, the dimensions of the surface through which dissolution and diffusion occur are smaller than those of the ACI sample collection plates, making it impossible to use the entire sample. This could create more problems, both analytically and technically. From an analytical perspective, lower concentrations mean reduced sensitivity. From a technical perspective, dividing the sample can lead to uneven particle distribution and possible sample loss due to extra handling.

The disadvantage of the paddle over the disc apparatus is the curvature at the bottom of the vessel, which does not coincide with the flat surface of the disk or membrane on which the particles are deposited. Thus, when using the disk, a “dead space” is created below it, which is largely isolated from the rest of the dissolution medium. This problem is typically resolved by using spacers that create space around the edge of the disk, allowing the medium to circulate [9,19]. Improved circulation of the medium is also assisted by higher rotation speeds and disk dimensions that do not exceed half the diameter of the vessel for dissolution testing. Since the diameter of the membrane and the disk holder are similar to the diameter of the compendial dissolution vessel, it is necessary to use a non-compendial vessel with a flat bottom. This reduces the volume of the dead space to a negligible value. Also, the vessel's diameter is slightly smaller than the official one, and the paddle position is slightly lower than that prescribed by the pharmacopeias. It is important to emphasize here that the lower position of the paddle is conditioned by the simultaneous need for it to be wholly immersed in the dissolution medium, whose volume was 300 mL. The detailed dimensions of the apparatus used are given in Figure 1. Sampling was performed from a point approximately 25 mm from the bottom of the vessel and 5 mm from the vessel wall. The reason for this is the low liquid level above the paddles and the narrow space between the rotating paddles and the vessel wall. Therefore, it was not possible to perform sampling from a location equivalent to that prescribed in the pharmacopoeias.

**Figure 4.**
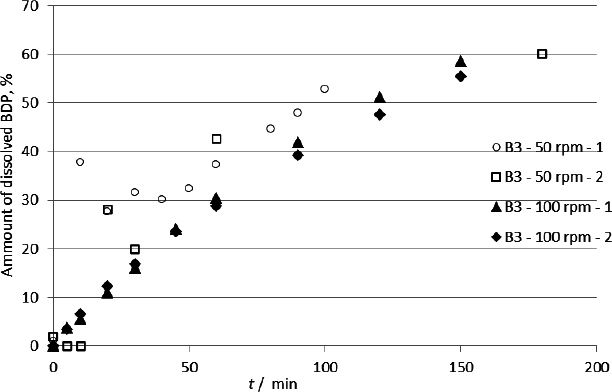
Effect of agitation (or paddle speed) on BDP dissolution rate from sample B3, designations 1 and 2 refer to two separate experiments

Pharmacopoeias do not provide a general guideline for the paddle rotational speed in the dissolution test. The values for the rotation speed are given in individual monographs of the products by the United States Pharmacopoeia, and the most commonly used speeds are 50, 75 and 100 rpm. Preliminary experiments were conducted with product B3, which contains BDP, at a speed of 50 rpm. At a paddle speed of 50 rpm, significant variations were observed between the results of repeated experiments, especially during the first hour of the test. By increasing the paddle speed to 100, the variation in the results disappeared, and the results became homogeneous and reproducible (Figure 4). Thus, the increase in media homogenization was achieved by increasing the rotation speed to 100 rpm.

All experiments were performed at a rotation speed of 100 rpm to standardize the assay. Although the dissolution medium used for SX testing did not contain SDS and theoretically should not have caused micelle formation and variation in results, the rotation speed was kept at 100 rpm for more intensive mixing.

Additional sources of variability in dissolution test results for inhalation products, related to sample size, include interactions with the membrane and limited solubility of the active substance in the selected medium. Problems with solubility are common for substances with low solubility, and the variability will be more pronounced as the sample size increases, where the saturation concentration of the solution is reached more quickly. Issues related to membrane interactions are typically the result of drug adsorption on the membrane.

Since the used membrane is highly hydrophilic, hydrophilic and ionic interactions would dominate the adsorption process, and these interactions would be more pronounced for substances that are highly soluble in water. Because membranes should have a specific adsorption capacity, the relative error is expected to increase as the sample size is reduced.

The influence of the amount of active substance on the dissolution rate was examined by varying the number of doses or device activations used to prepare the sample, depending on the reasoning, which factor is most problematic for high- or low-solubility substances. The number of doses was varied by ±50% relative to the selected reference sample size of 10 doses.

**Figure 5.**
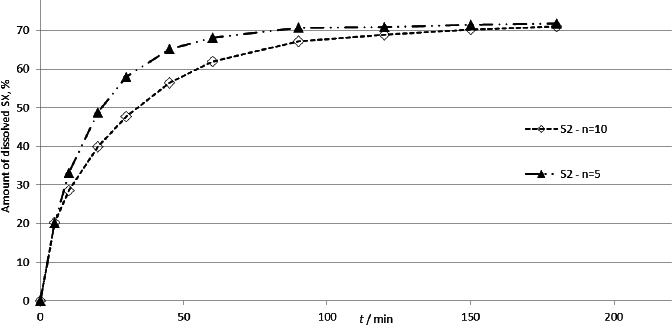
Dissolution profiles of salmeterol-xinafoate from samples containing 5 and 10 doses of product S2

Since SX has relatively good solubility, it was expected that the primary reason for the variation in the results could be adsorption on the membrane during the test. Therefore, the influence of drug loading was evaluated by comparing the dissolution rate of the sample (product S2, which contains only SX) with a smaller number of doses, *i.e.* 5 doses (Figure 5).

The difference between the dissolution profiles was examined using model-independent methods - calculating difference and similarity factors. Calculated factors suggest that the obtained results were comparable (*f*1 = 11.6, *f*2 = 53.8).

**Figure 6.**
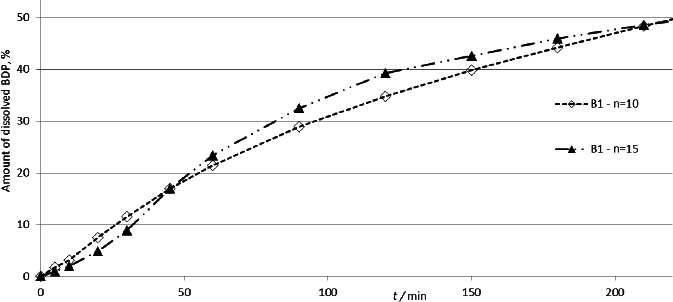
Dissolution profiles of beclomethasone dipropionate from samples containing 10 and 15 doses of product B1

A critical parameter for dissolution rate analysis of BDP is its solubility. Although the media volume was sufficient to satisfy the sink conditions, during the analysis, the BDP particles were found to be located in the narrow capillary space between the agar membrane and the metal plate of the cascade impactor. To diffuse further through the polysaccharide membrane, they must first dissolve in the small volume of liquid surrounding them in this capillary space, which can lead to rapid saturation and affect the results. The effect of higher concentrations was examined by comparing the drug dissolution profiles obtained with the standard sample, consisting of 10 activations, and the sample composed of 15 activations. The obtained profiles are presented in Figure 6.

No significant difference in BDP dissolution rate was observed between different sample sizes (*f*1 = 7.5, *f*2 = 78.0).

The absence of the influence of sample size on variation is important, as it allows the use of multiple doses, meaning that quantifying small amounts of active substance should be easier and can be carried out using conventional analytical methods, *i.e.* HPLC with UV-PDA detector.

**Figure 7.**
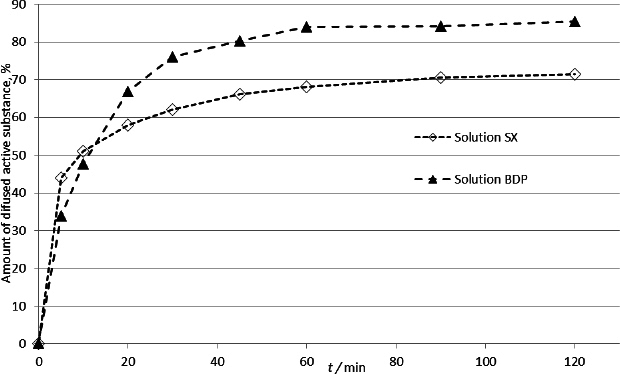
Diffusion profiles of SX and BDP from drug solutions

In addition to the investigated inhalation products, the diffusion profiles of the investigated active substances from solution have been recorded (Figure 7). A quantity of active substance (121.56 μg of salmeterol and 78.06 μg of BDP dissolved in dissolution medium) was transferred to an inverted ACI sample collection plate and carefully covered with a previously prepared agar membrane. The membrane was fixed with clips and immediately analysed using a procedure identical to the one developed for the dissolution method of inhalation products. In this manner, the selectivity of the experimental setup was tested. With this test, a "baseline" was obtained, which was not affected by the dissolution rate of solid particles of the active substance but only by the diffusion rate through the membrane. Additionally, the time deviation from the start of the test indicated a proper technical setup of the apparatus. The lack of an immediate release of the tested substance and its performance along the release profile curve plateau suggested that the apparatus system was effectively sealed. This shows that there is no significant leakage of the solution with the tested substance along the edges of the metal plate and the agar membrane, and that the increase in concentration originated from the substance that diffused through the agar membrane.

Figure 7 shows the diffusion profiles obtained by testing solutions of SX and BDP under the designed dissolution test setup.

There was a noticeable difference in the amounts of the tested substances detected in the medium at the end of the test. The proportion of SX diffusing through the membrane was 15 % lower than that of BDP. This may be due to the binding process of salmeterol to the membrane. Agar is a mixture of two polysaccharides: agaropectin and agarose. Agaropectin is composed of D-galactose, 3,6-anhydro galactose, D-glucuronic acid, pyruvic acid, and multiple ester-linked sulphate groups [45]. These acidic residues form potential binding sites for salmeterol base, which could explain the obtained results.

**Figure 8.**
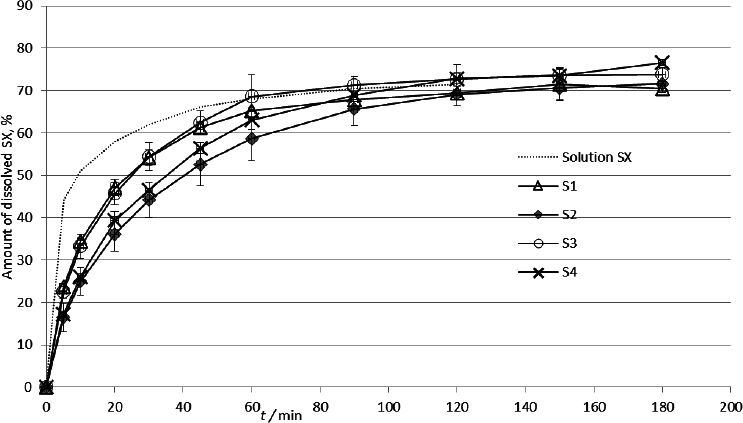
Dissolution profiles of SX samples (*n* = 3, mean ± SD)

The dissolution profiles of the SX formulations are presented in Figure 8. The shape and uniformity of the obtained profiles indicate a relatively rapid dissolution of the active substance, confirming the assumption that dissolution was not a limiting factor for SX release.

Comparison of the dissolution profiles using model-independent methods detected significant differences between products S2 and S4 with the reference Solution SX (Table 4). These differences were not detected for the products S1 and S3.

**Table 4.** Comparison of SX dissolution profiles from the tested products and the reference Solution SX using model-independent methods

Compared dissolution curves	*f*1	*f*2
S1 / Solution SX	13.8	52.9
S2 / Solution SX	**25.1**	**41.8**
S3 / Solution SX	13.3	51.9
S4 / Solution SX	**21.1**	**42.1**

Apart from the carrier gas, products S1, S2, and S3 do not contain any additional excipients. Product S4 additionally contains ethanol (as a cosolvent [46]) and soy lecithin (a surfactant that prevents particle agglomeration [47]).

A comparison of the dissolution profiles of the investigated products using model-independent methods did not reveal any differences in the dissolution profiles of the tested preparations, as shown in Table 5.

**Table 5.** Comparison of SX dissolution profiles from tested products using model-independent methods

Compared dissolution profiles	*f*1	*f*2
S1/S2	11.3	57.5
S2/S1	10.2	
S2/S3	11.8	56.2
S3/S2	13.4	
S1/S3	3.5	79.8
S3/S1	3.6	
S1/S4	9.3	62.2
S4/S1	8.8	
S2/S4	5.6	73.2
S4/S2	5.9	
S3/S4	8.1	64.0
S4/S3	7.6	

The presence of lecithin may explain the slight increase in the release rate of product S4 compared to product S2; however, it does not account for the faster release of products S1 and S3, which do not contain additional excipients except for the carrier gas and include an additional poorly soluble active substance - fluticasone propionate. The obtained dissolution curves of products S1 and S3 do not show significant differences compared to the release of the reference Solution SX. The presence of fluticasone propionate can lead to the formation of “combined particles” containing both active substances - SX and fluticasone propionate. Such a phenomenon was described by Theophilus *et al.* [48], who examined the degree of co-association of salmeterol and fluticasone propionate particles in combined MDI products compared to individual preparations. The creation of combined particles could lead to an increase in particle size, as detected in the tested samples. The median particle size (MMAD) values of SX in products S1 and S3, which were combined preparations, were almost twice as large as in products S2 and S4 containing only SX (Table 1). The creation of combined particles could also lead to the formation of hydrophobic parts due to the presence of fluticasone propionate, and together, this could result in a decrease in the release rate, which is contrary to the obtained results. The primary mechanism that can explain the somewhat faster release in products S1 and S3 is the difference in the contact angle *θ*. It has been shown that smaller particles tend to exhibit larger contact angles with the liquid, resulting in reduced wetting [49,50]. Since surface wetting is a prerequisite for drug dissolution [51], the increased contact angle for smaller particles in products S2 and S4 could explain the slower wetting and dissolution rates. This process contradicts the common understanding that, with decreasing size, the surface area in contact with the solvent increases, leading to more rapid dissolution. However, with very small particles, an interfering process occurs where decreasing particle size increases the wetting angle *θ* and consequently slows down the dissolution rate due to the hindered solvent-particle contact [49,50].

Although the mechanism mentioned above can explain the observed differences in SX dissolution rates, these differences were not significant when comparing the tested products using model-independent methods. The absence of significant differences between the obtained profiles (Tables 4 and 5) suggests that biopharmaceutical and pharmaceutical-technological differences between the tested products did not significantly impact SX dissolution, indicating that the dissolution process is not a rate-limiting factor for SX absorption.

The release profiles were compared using model-dependent methods, and the best correlation in the case of SX was obtained for the Higuchi kinetic model (Table 6), which describes the release of the substance depending on diffusion through the polymer matrix.

**Table 6.** Analysis of the dissolution profiles of SX with model-dependent models

Sample	Model			
	Zero order	First order	Hixson-Crowell	Higuchi
	*R* ^2^			
S1	0.5901	0.7191	0.6779	0.8291
S2	0.7436	0.8616	0.8257	0.9314
S3	0.6169	0.7453	0.7050	0.8490
S4	0.7373	0.8714	0.8305	0.9271

These results support the idea that the dissolution rate is not the limiting factor for the absorption of SX from inhalation preparations; rather, the limiting factor is diffusion, specifically through the polymer matrix of the agar membrane. No differences were found among the various SX formulations when compared using model-independent models. Based on all this, we can conclude that the dissolution of SX is relatively fast and that the dissolution rate is not a rate-limiting factor in the absorption of SX from inhalation products. A similar conclusion about the influence of dissolution rate on absorption was given by Eriksson *et al.* [52] when examining pulmonary absorption in models of isolated rat lungs and Caco-2 cell monolayers.

**Figure 9.**
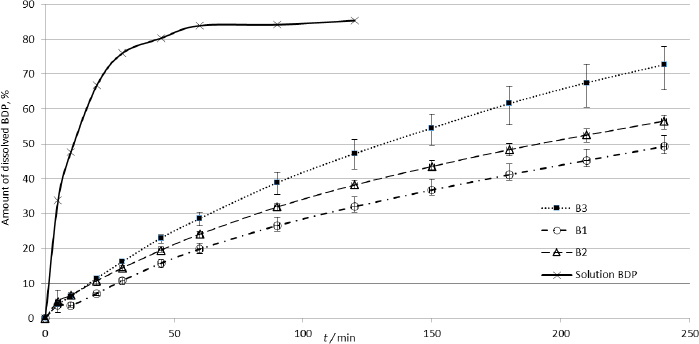
Dissolution profiles of BDP samples (*n* = 3, mean ± SD)

The dissolution profiles of the products containing BDP are shown in Figure 9.

The obtained profiles did not reach an equilibrium plateau within the experimental duration of the study (4 h). However, since most small undissolved particles are cleared from the lungs in less than 4 hours [53], and the absorption phase of BDP or the *t*_max_ of its active metabolite beclomethasone 17-monopropionate (BMP-17) is less than 4 hours [30,33], the duration of the test can be considered physiologically relevant.

The difference in drug release curves from the tested products and the solution suggests that dissolution is the rate-limiting factor for absorption. Similar observations have been made for other poorly soluble substances formulated as inhalation products [14,54]. The differences in the slopes obtained from the release curves indicate the potential differences in the rate and extent of BDP release from the tested preparations.

The obtained release profiles of the BDP products showed the highest correlation with the first-order kinetics and the Hixon-Crowell model (Table 7).

**Table 7.** Analysis of the dissolution profile of BDP by model-dependent methods - correlation coefficients (*R*^2^) of the dissolution profile with different release kinetics models

Sample	Model			
	Zero order	First order	Hixson-Crowell	Higuchi
	*R* ^2^			
B1	0.9733	0.9935	0.9883	0.9834
B2	0.9656	0.9931	0.9862	0.9110
B3	0.9750	0.9995	0.9971	0.9847

These results show that the BDP release was regulated by the concentration of the active substance (first-order kinetics) and the dissolution rate of BDP particles (Hixon-Crowell model), and it was less dependent on diffusion through the agar gel (Higuchi model).

Comparison of the dissolution profiles of BDP based on difference and similarity factors (*f*1 and *f*2 respectively) indicated statistically significant differences among the tested products (Table 8).

**Table 8.** Results of comparison of BDP dissolution curves from tested products using model-independent methods

Compared dissolution curves:	*f*1	*f*2
B1 - B2	16.9	61.4
B2 - B1	20.4	
B1 – B3	32.4	40.7
B3 - B1	47.9	
B2 – B3	19.2	50.5
B3 – B2	23.6	

Although the difference factor (*f*1) showed significant differences between all three formulations (*f*1 > 15), the similarity factor (*f*2) showed a difference only between products B1 and B3 (*f*2 < 50). The results indicate that the similarity factor is more adequate in detecting differences in the release profiles of BDP, a poorly soluble substance, and suggest that the proposed method can reveal differences in the dissolution profiles of various inhalation products containing the same active ingredient. This is particularly important when compared with the results of the aerodynamic particle size distribution and the differences in the amount of BDP deposited at the NGI stages. As can be seen in Table 1 and Figure 1, the largest difference was observed between products B1 and B3, which aligns with the results of the dissolution study.

A two-compartment pharmacokinetic model was created using GastroPlus™ software. The model was based on the results of *in vivo* studies published in the literature [30,32,33]. The obtained pharmacokinetic parameters were used in combination with the data defining the properties of the test substances (Table 9) to generate the expected *c*_p_-*t* curves data for intravenously (i.v.) and orally administered (p.o.) BDP and its main active metabolite, BMP-17.

**Table 9.** Data used for the creation of in silico model for BDP and BMP-17

	BDP i.v.	BMP-17	BDP p.o. / BMP-17
*M*_r_ / g mol^-1^	521.1 [41]	465.0 [55]	521.1 [41]
log *P*	1.30 [56]	2.46 [55]	1.30 [55]
p*K*a	-3.3 (base) 13.85 (acid) [41]	-3.3 (base) 13.85 (acid) [55]	-3.3 (base) 13.85 (acid) [41]
Solubility / mg mL^-1^ @pH 7	0.00208 [56]	0.04570 [57]	0.00030*
Diffusion coefficient, cm^2^ s10^-5^	0.7500	0.6072	0.5712
*V*_c_^a^ / L kg^-1^	0.080^f^	3.725 ^f^	6.000 ^f^
CL^b^/ L h^-1^	99.84 [30]	136.00 [30]	120.00 [41]
Dose / μg	1000	892	3570
Unbound fraction in plasma	0.05 [41]		
*t*_1/2_^c^ / h	0.47 [30]	2.42 [30]	4.28 [30]
*K*_12_^d^ / h^-1^	0.741^g^	1.403^g^	1.403^g^
*K*_21_^e^/ h-^1^	1.530^g^	1.997^g^	1.997^g^

^a^central volume of distribution;

^b^clearance;

^c^half-life;

^d^transfer rate constant from the central compartment (1) to the peripheral compartment (2);

^e^transfer rate constant from the peripheral compartment (2) to the central compartment (1);

^f^Optimized values;

^g^values predicted by GastroPlus™

To validate the model, the generated plasma profiles were compared with the equivalent curves from the *in vivo* studies. To achieve a better correlation between *in vivo* and in silico *c*_p_-*t* curves, the values given in the literature were not necessarily used; instead, the values were optimized within the range of reported results.

**Figure 10.**
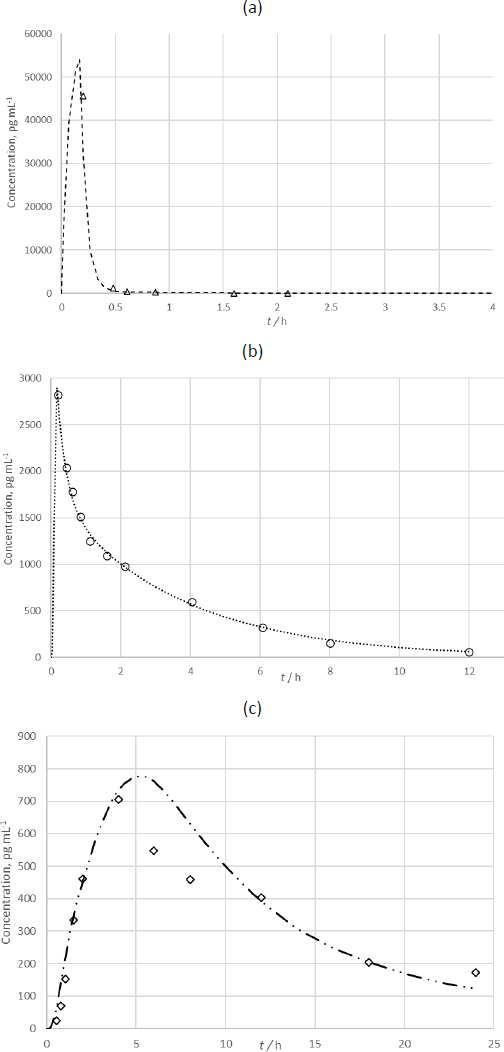
Simulated and *in vivo* concentration/time curves for a - BDP after i.v. application (-- - simulated; Δ - *in vivo*); b - BMP-17 after i.v. administration (··· - simulated; o - *in vivo*); c - BMP-17 after oral administration of BDP (-··- - simulated; ◊ - *in vivo*)

Figures 10a, 10b and 10c show a comparison of the generated *in silico* and *in vivo* data for intravenously (i.v.) and orally administered (p.o.) BDP and its main active metabolite, BMP-17. The correlation between the *in silico* and *in vivo* data is given in Table 10.

**Table 10.** Correlation between predicted (*in silico*) and *in vivo c*_p_-*t* curves of BDP

	BDP i.v aplication	BMP-17 after i.v. application of BDP	BDP/BMP-17 hybrid after oral application	BDP after application of the inhalation product	
				B1	B2
R^2^	0.821	0.997	0.893	n.a.*	0.99

*In the *in vivo c*_p_-*t* curve, there is only one point with a detectable amount of BDP in plasma, and no correlation can be established. Instead, the prediction error (PE) was calculated for the *c*_p_-*t* value at that time point (PE = 0.44 %)

A relatively low correlation (0.893) was obtained for the predicted *c*_p_-*t* profile after oral administration of BDP (Figure 10c and Table 10). In the *in vivo* study, following p.o. administration, BDP was not detected in the bloodstream; only its metabolite, BMP-17, was present. This posed a challenge that was addressed by creating a “hybrid” model that combined the “entry” parameters (parameters related to absorption) of BDP with the “exit” parameters (parameters related to elimination and distribution) of BMP-17. This approach simulated the absorption of BDP, its complete biotransformation in the liver, and the distribution and elimination of the metabolite, BMP-17. Considering that the oral route of administration will not be part of further testing and that this aspect of the simulation served as a form of cross-validation of the input parameters for the i.v. model, a correlation of 0.893 was deemed appropriate.

**Figure 11.**
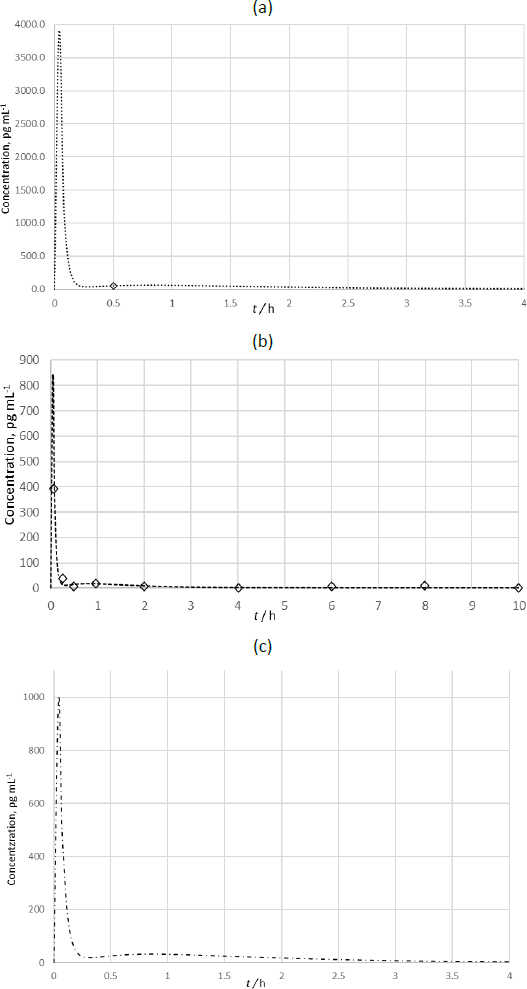
Simulated and *in vivo* concentration/time data for BDP after administration of inhalation products (a) B1 (··· - simulated; o – *in vivo*; Ref. 32); (b) B2 (---- - simulated; ◊ - *in vivo*; Ref. 33); (c) B3 (-·-·- - simulated)

The created model served as the basis for developing profiles of three inhalation products containing BDP. The model was supplemented with biopharmaceutical characteristics of each individual preparation. These characteristics are based on the size distribution of aerosol particles, which is assumed to provide insight into the localization of aerosol particle deposition in the respiratory tract and, consequently, the efficacy of the drug. The aerodynamic distribution of BDP particles was obtained using the Next Generation Impactor (NGI) (Copley Scientific, Nottingham, UK) according to the procedure outlined in the Ph. Eur. monograph on Aerodynamic Distribution of Fine Particles [2]. From these data, the pharmaceutical-technological parameters for the tested formulations were calculated using the CITDAS™ program, including MMAD and GSD, as well as the distribution of the active substance in the respiratory system. Distribution of BDP particles in the respiratory tract was calculated using a procedure described elsewhere [31]. Predicted *c*_p_-*t* curves are presented in Figure 11.

All three predicted *c*_p_-*t* curves had a similar shape. The presence of a second peak at 1 hour is noticeable. This peak is significantly smaller than the first peak and can be attributed to secondary absorption through the gastrointestinal tract, while the first, main peak is attributed to absorption through the lungs. Although the presence of this peak contradicts the literature data on the oral absorption of BDP (28), it was detected in a study that monitored the pharmacokinetics of BDP after respiratory administration of preparation B2 [33]. These discrepancies can be attributed to differences in the characteristics of the subjects (such as gender, age, body weight, and possibly race), as well as variations in analytical procedures.

The correlation between the simulated and *in vivo c*_p_-*t* curves of BDP for preparation B2 is 0.99, indicating excellent agreement. The main difference, also seen in the i.v. administration of BDP (Figure 10a) occurs in the first 2 data points, attributed to the lack of initial sampling points, which affects *c*_max_, *t*_max_, and AUC values. The correlation for preparation B1 couldn't be calculated due to the presence of only one measurable BDP concentration point after respiratory administration. Additionally, the first sampling was taken 30 minutes post-administration, missing the initial peak of *c*_max_, which occurs within the first 4 minutes. This resulted in a large portion of the area under the curve going undetected, despite the PPE for this point being low at 0.44 %. Due to insufficient *in vivo* data for preparation B3, a comparison with the in silico predicted *c*_p_-*t* curve was not possible.

The obtained curves of the dissolution profiles of BDP (Figure 9) do not reach a plateau with maximum release during the experiment. The primary parameter that distinguishes them is their slope. The slopes of the release curves were compared with the pharmacokinetic parameters obtained from the developed in silico models (Table 11).

**Table 11.** Pharmacokinetic parameters obtained from the developed *in silico* models

	B1	B2	B3
*k*_a_^a^ / h^-1^	44.01	38.43	37.22
*k*_10_^b^ / h^-1^	6.79×10^-3^	6.18×10^-3^	2.53×10^-3^
*c*_max_^c^ / ng mL^-1^ (mg of dose)^-1^	12.00	9.45	6.66
*t*_max_^d^ / h	0.04	0.04	0.04
*t*_1/2_ / h	2.42×10^4^	1.77×10^4^	2.77×10^4^
Slope of dissolution curves, % min^-1^	0.204	0.230	0.303

^a^absorption constant

^b^elimination constant

^c^maximum concentration in plasma

^d^time in which *c*_max_ was attained;

A statistical correlation parameter (determination coefficient, *R*^2^) between pharmacokinetic parameters and the slope of dissolution curves (Table 11) was determined by linear regression analysis. The results of the obtained correlations are presented in Table 12.

**Table 12.** Correlation between pharmacokinetic parameters obtained by the *in silico* method and the slope of the dissolution curve of inhalation products containing BDP.

	*R* ^2^
*k*_a_ / h^-1^	0.6567
*k*_10_ / h^-1^	0.9855
*c*_max_ μg mL^-1^ (mg of dose)^-1^	0.9415
*t*_1/2_ / h^-1^	0.3398

A good correlation between the slope of the dissolution curve of the BDP was observed with *c*_max_, but not with *t*_1/2_. Due to the same value of *t*_max_ for all 3 products, correlation for that pharmacokinetic parameter was not calculated.

A very good correlation was noticeable with k_10_ and a lesser one with *k*_a_ (Table 12). Since *k*_10_ defines the process of drug elimination from the primary compartment, there is no basis for its connection with the parameters that define the dissolution rate, which is a prerequisite for absorption. On the other hand, flip-flop kinetics could explain such correlations.

In the flip-flop kinetics, there is an “inversion” of the absorption and elimination constants estimated from the *c*_p_-*t* curve. This phenomenon usually occurs in products with a prolonged or slow release, where the absorption constant is significantly lower than the elimination constant. Thus, in this case, the apparent elimination constant (*k*_10_) would represent the absorption constant, since the possible elimination rate is significantly higher than the absorption rate, and the elimination itself would occur at the maximum rate at which the active substance is absorbed, or the rate defined by the absorption constant of BDP. Therefore, the descending part of the *c*_p_-*t* curve, from which the elimination constant is usually determined, is related to the absorption constant [58,59]. To confirm that a particular preparation underwent flip-flop kinetics, a comparison with the calculated elimination constant during i.v. administration of the test substance is usually used. If the elimination constant obtained after i.v. administration is close to that obtained in the preparation where flip-flop kinetics is suspected; this is considered evidence that it is present in that case. Conversely, the similarity of the elimination constant in i.v. administration, and in products where the flip-flop kinetics are suspected, it indicates that flip-flop kinetics do not occur in that case [60]. Elimination and absorption constants calculated from the *c*_p_-*t* curves obtained from the developed *in-silico* models are given in Table 9. The elimination constant based on the *in-silico* model of BDP i.v. administration was 17.15 h^-1^. The obtained value was much closer to the values of the k_a_ calculated for administration via the respiratory tract (from 37.22 to 44.01 h^-1^) than to the values obtained for the elimination constant (from 6.79×10^-3^ to 2.53×10^-3^ h^-1^), which indicates the possibility of the presence of flip-flop kinetics.

The “interchange” of the absorption and elimination constants is characteristic in cases where the release and/or dissolution of the active substance is slowed down. This may be due to the low solubility of the active substance or the modified release formulation. Although BDP has low solubility, absorption is further slowed by the effect of the extremely small volume of pulmonary fluid available for dissolution and by mechanisms of particle removal from the respiratory tract, which further reduces the ratio of absorbed active substance [61]. Based on this, it can be inferred that the calculated *k*_10_ is a measure of the absorption rate. Thus, the correlation with the slope of the release rate curve becomes logical and significant for establishing the claim about the *in vivo* predictability of the developed method for dissolution testing of inhaled products containing BDP.

The obtained correlation was achieved at two points: between *k*_a_ and the slope of the dissolution curve and *c*_max_ and the slope of the dissolution curve of the active substance from the preparation, which corresponds to the multiple Level C correlation as defined in the USP. All the predicted pharmacokinetic parameters were obtained as a result of *in silico* simulations, which showed good agreement with the *in vivo* data from the literature.

The obtained correlation indicates that the dissolution rate of BDP in the tested dissolution system strongly correlated with the data obtained by the *in silico* method. It would be optimal to establish a correlation between the *in vitro* and *in vivo* data; however, the lack of published data on the pharmacokinetic profiles of inhalation products containing BDP makes such a comparison challenging. Also, the selection of inadequate sampling sites at the beginning of the sampling procedures in *in vivo* studies significantly impacted the obtained pharmacokinetic parameters. This is especially visible in the calculation of *t*_max_ and *c*_max_, and thus also on k_a_. These shortcomings were addressed by developing an *in silico* model for BDP. Additionally, using this approach, the *c*_p-*t*_ curve can be derived for new BDP inhalation products based on their biopharmaceutical properties, thus generating sufficient data to examine possible correlations.

## Conclusions

The *in vitro* method was developed to evaluate the dissolution rate of the preparation for inhalation. The method's settings (dissolution medium, volume of medium available for particle dissolution, fine particle fraction used, and surfactant usage) were adjusted to loosely mimic conditions in the respiratory tract without significantly increasing the method's complexity or reducing its acceptability. The developed method for determining the dissolution rate revealed fast dissolution and did not detect a difference between inhalation products containing SX, a model substance with relatively good solubility. That is in accordance with the proposed iBCS [62], where SX is placed in group III and close to the border of group I. Both of those groups are composed of substances that have rapid dissolution. Analysis of the dissolution profile of SX using model-dependent methods did not indicate that dissolution was a rate-limiting process in the release of the active substance. A developed dissolution method detected differences in the dissolution profiles of the analysed inhalation products containing BDP using model-independent methods. BDP is classified in group II of iBCS, which is characterized by dissolution-dependent absorptive and non-absorptive clearance and retention [62]. Analysis of dissolution profiles using model-dependent methods indicated that dissolution is the rate-limiting factor for absorption of BDP. Therefore, *in silico* ADME model of BDP was developed and pharmacokinetic constants (*c*_max_, *t*_max_, *k*_a_, *k*_10_) linked to each tested inhalation product were calculated. A good correlation was found between the slope of the dissolution curve and *k*_a_ (represented by *k*_10_ in the case of flip-flop kinetics) and *c*_max_, indicating the clinical relevance of the developed dissolution method.

These results strongly support the importance of the dissolution method in quality control and bioequivalence testing of inhalation products containing Class II and IV drug substances. Considering the reawakened interest of regulatory agencies, this method can serve as a first step toward a generally accepted methodology for conducting dissolution testing of inhalation products.
